# *Salacca zalacca* extract's antiaging effect on aging genes, protein levels, and apoptosis in UV-induced fibroblast cells

**DOI:** 10.1016/j.jtumed.2025.05.005

**Published:** 2025-06-09

**Authors:** Wahyu Widowati, Dani Dani, Vera Vera, Teresa L. Wargasetia, Fanny Rahardja, Fen Tih, Philips Onggowidjaja, Rita Tjokropranoto, Fadhilah H. Zahiroh, Rizal Azis, Didik Priyandoko, Wahyu Surakusumah, Dhanar S. Hadiprasetyo

**Affiliations:** a Faculty of Medicine, Maranatha Christian University, Bandung, West Java, Indonesia; b Bimolecular and Biomedical Research Center, Aretha Medika Utama, Bandung, West Java, Indonesia; c Biomedical Engineering, Department of Electrical Engineering, Faculty of Engineering, Universitas Indonesia, Depok, West Java, Indonesia; d Biology Study Program, Faculty of Mathematics and Science Education, Universitas Pendidikan Indonesia, Bandung, West Java, Indonesia; e Faculty of Pharmacy, Universitas Jenderal Achmad Yani, Cimahi, West Java, Indonesia

**Keywords:** مضاد للشيخوخة, مضاد للأكسدة, حمض الكلوروجينيك, فاكهة الثعبان, الأشعة فوق البنفسجية, Antiaging, Antioxidant, Chlorogenic acid, *Salacca zalacca*, Ultraviolet

## Abstract

**Objectives:**

Ultraviolet (UV) exposure can hasten the aging process of the skin. The use of chemicals for anti-aging has long-term adverse effects. The natural ingredients of snake fruit (*Salacca zalacca* L.) are known to have bioactive properties such as polyphenols, flavonoids, chlorogenic acid, and caffeic acid which have antiaging potential. The study aims to ascertain the potential of *S. zalacca* Extract (SZE) as an antiaging agent by *in vitro* assay.

**Methods:**

The SZE compound content was analyzed by LC-MS/MS. SZE viability test on human skin fibroblast (BJ) cells was carried out using the WST assay. BJ cells were UV-induced as a cell model of premature aging. SZE 6.25, 12.5, and 25 μg/mL were administered to UV-induced BJ cells. The gene expression of *COL1A1, MMP-1, FGF-2,* and *GPX-1* were analyzed by quantitative Real-Time PCR. Elastin (ELN), Hyaluronidase (HAse), Cyclooxigenase-2 (COX-2), 8-Hydroxydeoxyguanosine (8-OHdG), and Melatonin (MT) protein levels were analyzed by ELISA assay. The apoptosis of BJ cells was analyzed using flow cytometry. One-way ANOVA in SPSS Software was used for statistical analysis.

**Results:**

Treatment with SZE increased *COL1A1*, *FGF-2*, and *GPX-1* gene expression and also decreased *MMP-1* gene expression. SZE also increased ELN and MT levels in UV-induced BJ cells. After SZE treatment, the protein levels of HAse, COX-2, and 8-OHdG decreased compared to the positive control. SZE also succeeded in maintaining the lives of BJ cells and reducing apoptosis in BJ cells.

**Conclusions:**

SZE has the potential to be an antiaging agent by *in vitro* assay.

## الملخص

**أهداف البحث:**

يُمكن أن يُسرّع التعرض للأشعة فوق البنفسجية شيخوخة الجلد. ولاستخدام المواد الكيميائية المُضادة للشيخوخة آثار جانبية طويلة المدى. ومن المعروف أن المكونات الطبيعية لفاكهة الثعبان (سالاكا زالاكا) تتميز بخصائص حيوية نشطة، مثل البوليفينولات والفلافونويدات وحمض الكلوروجينيك وحمض الكافيين، والتي تُعدّ مُضادة للشيخوخة.

**طرق البحث:**

تهدف الدراسة إلى تحديد قدرة مستخلص فاكهة الثعبان كمضاد للشيخوخة من خلال اختبار مُختبري. وقد حُلِّل محتوى المستخلص بواسطة الاستشراب السائل مع قياس الطيف الكتلي الترددي. وأُجري اختبار قابلية المستخلص للبقاء على خلايا الأرومة الليفية الجلدية البشرية باستخدام اختبار WST. وقد استُحثت خلايا الأرومة الليفية بالأشعة فوق البنفسجية كنموذج خلوي للشيخوخة المُبكرة. وأُعطيت 3 جرعات من مستخلص فاكهة الثعبان (6.25 و12.5 و25 ميكروغرام/مل) لخلايا الأرومة الليفية المُحرضة بالأشعة فوق البنفسجية. تم تحليل التعبير الجيني لجينات COL1A1 وMMP-1 وFGF-2 وGPX-1 باستخدام تفاعل البوليميراز المتسلسل الكمي في الوقت الحقيقي. وتم تحليل مستويات بروتينات الإيلاستين، والهيالورونيداز، وسيكلوكسيجيناز-2، و8-هيدروكسي ديوكسي غوانوزين، والميلاتونين باستخدام اختبار إليزا. وتم تحليل موت الخلايا المبرمج لخلايا الأرومة الليفية باستخدام قياس التدفق الخلوي. واستُخدم تحليل التباين أحادي الاتجاه للتحليل الإحصائي.

**النتائج:**

أدى العلاج بـمستخلص فاكهة الثعبان إلى زيادة التعبير الجيني لجينات COL1A1 وFGF-2 وGPX-1، وانخفاض التعبير الجيني لجين MMP-1. كما زاد المستخلص من مستويات الإيلاستين والميلاتونين في خلايا الأرومة الليفية المُستحثة بالأشعة فوق البنفسجية. وبعد العلاج بـمستخلص فاكهة الثعبان، انخفضت مستويات بروتينات الهيالورونيداز وسيكلوكسيجيناز-2 و8-هيدروكسي ديوكسي غوانوزين مقارنةً بمجموعة التحكم الإيجابية. نجح مستخلص فاكهة الثعبان أيضًا في الحفاظ على حياة خلايا الأرومة الليفية وتقليل موت الخلايا المبرمج فيها.

**الاستنتاجات:**

يمتلك مستخلص فاكهة الثعبان القدرة على أن يكون عاملًا مضادًا للشيخوخة من خلال اختباره في المختبر.

## Introduction

Solar ultraviolet (UV) radiation has a substantial negative influence on human health. They can damage DNA by penetrating the layers of skin and increasing the risk of skin cancer as well as hastening the aging process. In particular, those who reside in regions with high UV exposure levels should be cautious about this.^1^, ^2^, ^3^ To overcome premature aging of the skin, many skin care products are used that contain certain chemical compounds such as retinol and hydroxy acids which provide good short-term results but can cause long-term side effects.^4^ Therefore, there is an urgent need to search for natural ingredients that have the potential to be safer and more sustainable substitutes in the field of antiaging skin care therapy. Many natural ingredients exhibit antioxidant and anti-aging bioactivity, thus having the potential to reduce the manifestations of aging on the skin. Snake fruit has emerged as a promising natural ingredient for further investigation due to its potential bioactivities. Several studies show that snake fruit (*Salacca zalacca* L.) is known to have quite strong phenolic content and antioxidant activity.^5^^,^^6^ Additional research shows that snake fruit exhibits important antioxidant potential through its ability to capture 2,2-diphenyl-1-picrylhydrazyl (DPPH) radicals,^7^ Ferric Reducing Antioxidant Power (FRAP) activity^3^ and *S. zalacca* fruit extract has antiaging activity via elastase inhibition.^3^

Several studies have succeeded in explaining the cellular pathways involved in the skin aging process due to UV radiation, which is essential to determine targets in antiaging therapy.^8^^,^^9^ UV radiation can disrupt the control of the *Matrix Metalloproteinase-1 (MMP-1)* gene which is involved in *Type 1 Collagen* (*COL1*) degradation.^10^ MMP activation also interferes with the activity of collagen (COL) synthesis proteins, especially the *Collagen Type I Alpha 1 chain (COL1A1).* UV exposure can also disrupt the regulation of the *Fibroblast Growth Factor-2 (FGF-2)* gene which has a significant impact as a growth factor in the process of proliferation, differentiation, and repair of fibroblast cells.^11^

UV radiation stimulates the generation of *Reactive Oxygen Species* (*ROS*) in the skin. Destroying DNA, proteins, and lipids which can accelerate the skin aging process. The enzyme *Glutathione Peroxidase-1* (*GPX-1*) plays an important function in fighting ROS caused by UV radiation by degrading ROS so that it can be a target for antiaging therapy.^12^ UV radiation also impacts the apoptotic pathway. UV radiation can induce increased apoptosis which results in a decrease in living fibroblast cells in the dermis resulting in decreased tissue function.

UV radiation can also significantly affect the expression of aging-related proteins. One of the main proteins affected is elastin (ELN), which is essential for maintaining skin elasticity. UV exposure causes ELN degradation, primarily through the activation of Matrix Metalloproteinases (MMPs), particularly elastase, which is exacerbated by oxidative stress. This degradation contributes to the loss of skin firmness and wrinkle formation. In addition, UV radiation also affects melatonin (MT) levels, a well-known antioxidant that protects cells from oxidative stress. This decrease in MT, combined with increased hyaluronidase (HAase) activity, further complicates the skin's response to UV damage. Hyaluronidase activity can increase after UV exposure, leading to accelerated breakdown of hyaluronic acid, which is essential for skin hydration. The resulting decrease in skin hydration can worsen visible signs of aging and compromise the skin's structural integrity. In addition to these changes, UV radiation increases the expression of Cyclooxigenase-2 (COX-2), an enzyme that plays a key role in the inflammatory response. Increased COX-2 activity promotes inflammation and contributes to skin damage. The cumulative effect of these changes is reflected in increased levels of 8-hydroxy-2′-deoxyguanosine (8-OHdG), a biomarker indicating oxidative DNA damage. Increased levels of 8-OHdG indicate increased genomic instability and increased risk of mutations, which may lead to skin carcinogenesis. This association underscores the critical impact of UV radiation on skin health and the importance of protective measures against UV exposure.

Further research is required to evaluate the anti-aging properties of *S. zalacca* extract (SZE) and learn more about its molecular mechanisms by investigating its effects on the expression of aging-related genes and proteins. This research aimed to assess the efficacy of SZE as a potential antiaging agent by conducting *in vitro* experiments on BJ cells by UV induction by measuring gene expression of *COL1A1, MMP-1, FGF-2* and *GPX-1,* the protein level of ELN, MT, HAse, COX-2, and 8-OHdG, the percentage of apoptotic cells. The anticipated outcomes of this study are expected to yield novel perspectives on the safe and efficient exportation of natural anti-aging constituents.

## Materials and Methods

### Preparation of *S. zalacca* extract

The collection of botanical specimens was carried out at Bogor, located in the province of West Java, Indonesia. Subsequently, the plant material was recognized by the Biology Department of the School of Life Sciences and Technology at the Bandung Institute of Technology. The *S. zalacca* fruit undergoes a process of cleansing, seed separation, drying, and subsequent fine grinding into a powdered form. The powder was immersed in a solution of 70 % ethanol. After filtration, the SZE was evaporated at 40 °C, until it became a paste. Lactose was then added to the paste.^13^ The SZE underwent processing at PT FAST (Depok, Indonesia) following Good Manufacturing Practices and CoA No. Batch 001.11.23. ERTL.01.

### LC/MS-MS

The compound content contained in SZE was analyzed using LC/MS-MS. A column with Hypersil Glod was used for analysis. With electrospray ionization (ESI), the TSQ Quantum Access MS/MS Triple Q mass spectrometer is operated with a positive charge.^13^^,^^14^ Caffeic acid (Chengdu, BP0300), epicatechin (Chengdu, BP0538), chlorogenic acid (Chengdu, BP0345), ferulic acid (Chengdu, BP0587), naringenin (Chengdu, BP0981), apigenin (Chengdu, BP0177), and gallic acid (Chengdu, BP0608) is used as the standard.

### Viability assay

Human Fibroblast (BJ) Cells (ATCC® CRL-2522™) were obtained from Aretha Medika Utama, Bandung, Indonesia. The cells were cultured in a Minimum Essential Medium (MEM) (Biowest, L0416-500) as basal and added Fetal Bovine Serum Premium (FBS) (Biowest, S181B-500), Antibiotic-Antimycotic (ABAM) (Biowest, L0010-100), Gentamicin (Gibco, 15750060), Amphotericin B (Biowest, L0009-050), MEM Vitamins 100× (Biowest, X0556-100) and MEM Non-essential amino acids 100× (Biowest, X0557-100).^15^ The cells were then incubated in an incubator (Thermo IH3543) at 37 °C with 5 % CO_2_. After a 24-h incubation period, the viable cell count was determined using a hemocytometer (Neubauer). Briefly 1 × 10^4^ cells were seeded in 96-well plates (Costar 3596).^16^ Cells were cultured for 24 h at 37 °C. The culture medium was replaced with 90 μL new culture medium, and 10 μL SZE (3.13, 6.25, 12.5, 25, 50, 100, and 200 μg/mL). The cells were incubated for 24-h. Briefly, 10 μL of CCK-8 buffer (Elabscience, E-CK-A362) was added and incubated for 3-h. Spectrophotometry (Multiskan GO Thermo Scientific 51119300) at a wavelength of 450 nm was used to measure the absorbance.^6^^,^^16^

### Treatment SZE on UV-induced human fibroblast cells

Briefly, 10^6^ cells at 80 % confluence were exposed to UV light in a six-well plate for 75 min at 37 °C and 5 % CO_2_. After being exposed to UV light, then treated with SZE at concentrations of 6.25, 12.5, and 25 μg/mL for four days, cells were harvested using 0.25 % trypsin-EDTA (Gibco, 25200072).^6^

### qRT-PCR assay

RNA was extracted using TRI Reagent (Zymo Research, R2050-1-200) and Direct -zol ™ RNA Miniprep Plus (Zymo Research, R2073). Estimation of the total RNA yield was conducted using a spectrophotometer (Multiskan GO Thermo Scientific 51119300) at 260 and 280 nm. SensiFAST cDNA Synthesis Kit (Bioline, BIO-65054) was used to synthesize cDNA from RNA with a three-stage protocol, priming, reverse transcription, and RT inactivation. PCR amplification was conducted using the AriaMx RT-PCR System (Agilent, G8830A). The qPCR conditions encompassed an initial pre-denaturation step at 95 °C for 5 min, followed by 40 cycles of qPCR, involving denaturation at 95 °C for 50 s, annealing at 54 °C (*MMP-1*), 57 °C (*COL1A1*), 58 °C (*GAPDH* and *FGF-2*) and 59 °C (*GPX-1*) for 50 s, and elongation at 72 °C for 50 s.^3^ The housekeeping gene *GAPDH* served as the internal control. The primers used for real-time PCR are outlined in Table 1.

**Table 1:** The primary sequence of genes used.

Gene	Primary Sequence (5′ - 3′)	Annealing (°C)	Cycle	References
*FGF-2* (*Homo sapiens*)	GGCTTCTTCCTGCGCATCCA	58	40	NM_002006.6
	GCTCTTAGCAGACATTGGAAGA			
*GPX-1* (*Homo sapiens*)	CCAAGCTCATCACCTGGTCT	59	40	NM_001329455.2
	TCGATGTCAATGGTCTGGAA			
*MMP-1* (*Homo sapiens*)	CTGAAGGTGATGAAGCAGCC	54	40	NM_001145938.2
	AGTCCAAGAGAATGGCCGAG			
*COL1A1* (*Homo sapiens*)	CGGCTCCTGCTCCTCTTAG	57	40	XM_054315083.1
	CACACGTCTCGGTCATGGTA			
GAPDH (*Homo sapiens*)	GGGCTGCTTTTAACTCTGGT	58	40	NM_001357943.2
	TGGCAGGTTTTTCTAGACGG			

### ELISA assay

ELISA testing follows the protocol according to KIT, namely Human ELN Kit (Elabscience, E-EL-H1163), Human HAse Kit (Elabscience, E-EL-H2201), Human COX-2 Kit (E-EL-H5574), 8-OHdG Kit (Elabscience, E-EL-0028), and Human MT Kit (Elabscience, E-EL-H2016). The protocol was done based on the manual kit and the measurement of absorbance was conducted via a microplate reader (MultiskanTM Spectrophotometer).^17^

### Apoptosis assay

The apoptosis percentage (live cell, necrosis, early and late apoptosis) of BJ cells was analyzed with prior research with modifications.^18^ Each treatment group was analyzed using the Apoptosis Kit (Elabscience, ECK-A211). The treated cells were harvested and rinsed using 1 mL of FACS buffer and centrifuged at 1600 rpm for 5 min. The cell pellet was then added with 500 μL of Annexin binding buffer. Then the samples were stained using 5 μL Annexin V-FITC/PI and 5 μL Propidium Iodide Per Cp. Cy5 (30 min incubation, 4 °C (dark room)). Cell apoptosis was measured using MACSQuant 10 flow cytometry.

### Statistical analysis

The data was statistically analyzed with SPSS 23.0 (SPSS Inc; USA) using one-way ANOVA, followed by Tukey HSD post hoc test for normally distributed and homogeneous data, Mann Whitney post hoc test was performed for normally and not normally distribution data but not homogeneous data. P value ≤ 0.05 was considered as the significant value of the data. Data were visualized as mean ± standard deviation from three replications in histograms created in the GraphPad Prism application (version 8.0.244).

## Results

### Compound analysis in SZE by LC/MS-MS

The results of the compound content analysis in SZE using LC/MS-MS can be seen in Supplementary Figure 1. LC-MS/MS analysis showed that SZE contained several compounds including chlorogenic acid, epicatechin, naringenin, and apigenin.

### Cell viability test

**Figure 1:**
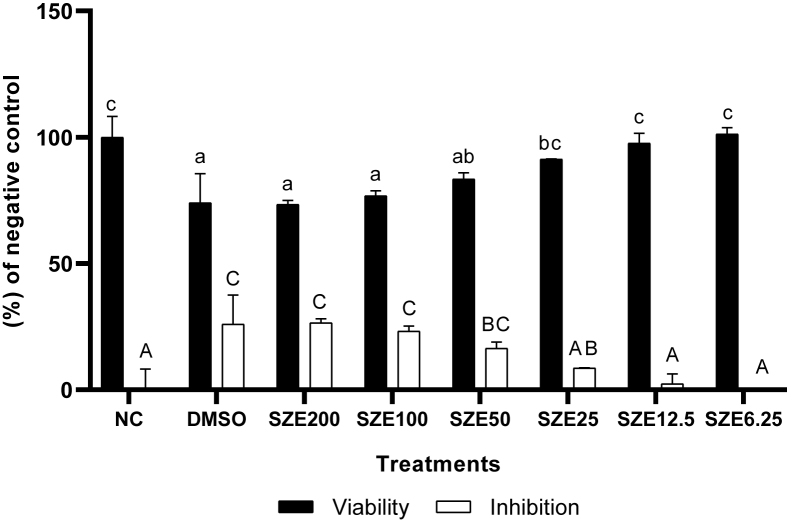
Effect of SZE Concentrations toward Viability and Inhibition Percentage in BJ Cells. ∗ NC: negative control (normal cells); DMSO: Positive Control + DMSO 1%; SZE: treatment with SZE with different concentrations (200, 100, 50, 25, 12.5, 6.25 μg/mL); All the differences capital letters (A, AB, BC, C) and small letters (a, ab, bc, c) show a significant difference among treatments based on the Mann Whitney post hoc test (p < 0.05).

The results show that SZE has no significant cytotoxic effect on BJ cells, as the highest concentration resulted in only 26.56 % inhibition (Figure 1). For further testing, a concentration range was selected with cell viability results above 90 %, namely 25, 12.5, and 6.25 μg/mL.

### Effect of SZE concentrations toward *COL1A1*, *FGF-2*, *GPX-1* and *MMP-1* genes expression in UV-induce BJ cells

**Figure 2:**
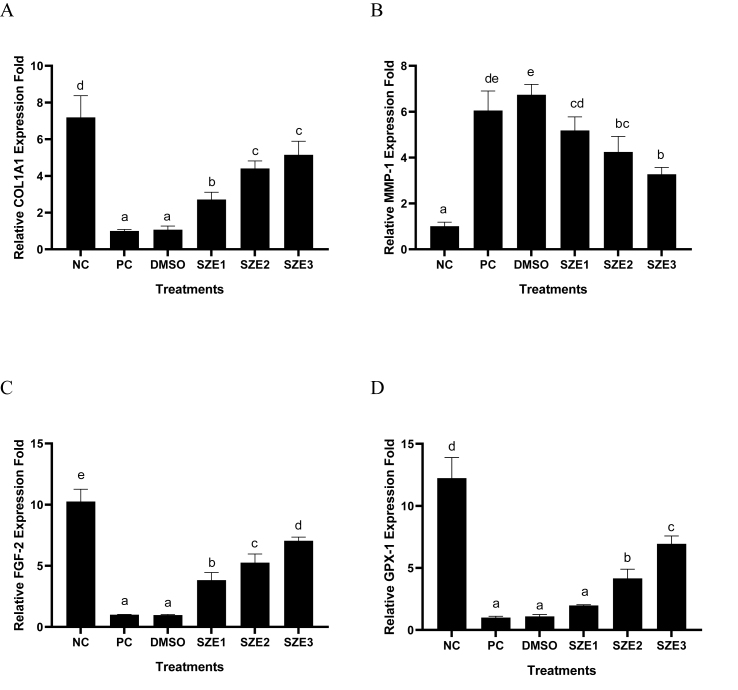
Effect of SZE Concentrations toward *COL1A1, MMP-1*, GPX-1, and *FGF-2* Genes Expression in UV-induced BJ Cells. ∗ NC: negative control; PC: positive control (UV-induced cells); DMSO: PC + DMSO 1 %; SZE1: PC + SZE 6.25 μg/mL; SZE2: PC + SZE 12.5 μg/mL; SZE3: PC + SZE 25 μg/mL The Tukey Post Hoc test (p < 0.05) indicates a significant difference between treatments for all letters in *COL1A1* (A) and *MMP-1* (D). The different letters in GPX-1 (B) *FGF-2* (C) reveal a significant difference by Mann Whitney test (p < 0.05).

The results of gene expression in UV-induced BJ cells treated with SZE can be seen in Figure 2. UV induction in BJ cells can influence BJ cell gene expression as seen by the significant differences between the Normal Cells (NC: UV-uninduced cells: I) and Positive Control (PC: UV-induced cells: II) groups in each gene expression. Treatment with SZE increased significantly (p < 0.05) the expression of the *COL1A1*, *GPX-1*, and *FGF-2* genes. SZE also significantly reduced (p < 0.05) *MMP-1* gene expression in UV-induced BJ cells. The most active concentration for increasing *COL1A1*, *GPX-1*, and *FGF-2* gene expression, as well as decreasing *MMP-1* was a concentration of 25 μg/mL.

### Effect of SZE concentrations toward ELN and HAse proteins levels in UV-induced BJ cells

**Figure 3:**
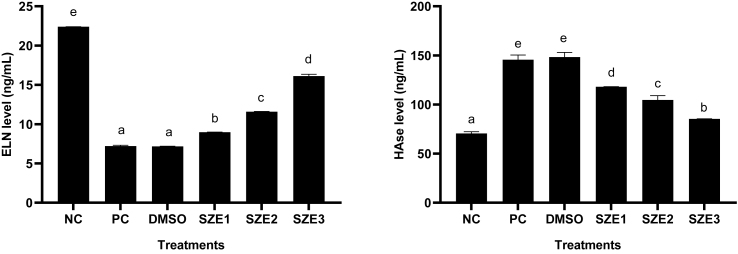
Effect of SZE Concentrations toward ELN and HAse levels in UV-Induced BJ Cells. ∗ NC: Negative Control; PC: Positive Control (UV-induced cells); DMSO: PC + DMSO 1 %; SZE1: PC + SZE 6.25 μg/mL; SZE2: PC + SZE 12.5 μg/mL; SZE3: PC + SZE 25 μg/mL. The Mann Whitney test (p < 0.05) indicates a significant difference in ELN letters (A), HAse (B) between treatments.

The results of ELN and HAse protein levels in UV-induced BJ cells treated with SZE are presented in Figure 3. The results show that UV induction could decrease ELN and increase HAse levels in BJ cells. SZE treatment increased ELN and decreased HAse levels in UV- induced BJ cells. SZE treatment with a concentration of 25 μg/mL was the most active in increasing ELN and reducing HAse levels than other concentrations.

### Effect of SZE concentrations on COX-2, 8-OHdG, and MT levels in UV-induced BJ cells

**Figure 4:**
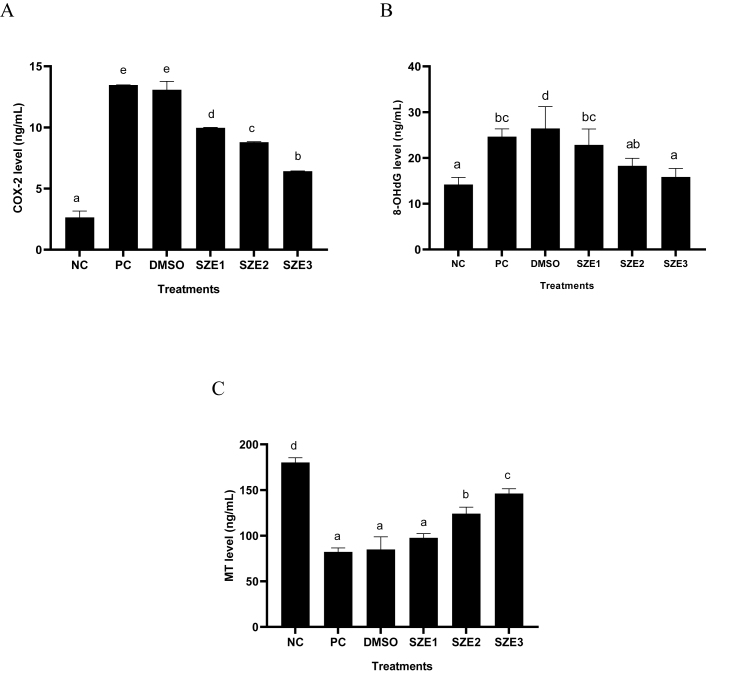
Effect of SZE Concentrations toward COX-2, 8-OHdG, and MT Levels in UV-Induced BJ Cells. ∗ NC: Negative Control; PC: Positive Control (UV-induced); DMSO: PC + DMSO 1 %; SZE1: PC + SZE 6.25 μg/mL; SZE2: PC + SZE 12.5 μg/mL; SZE3: PC + SZE 25 μg/mL. The Mann Whitney test (p < 0.05) revealed a significant difference between treatments in COX-2 level. The Tukey Post Hoc test (p < 0.05) revealed a significant difference in between treatments in 8-OHdG and MT level.

The results of COX-2, 8-OHdG, and MT levels in UV-induced BJ cells treated with SZE are presented in Figure 4. The results show that UV induction increased COX-2 and 8-OHdG proteins and decreased MT significantly (p < 0.05) in BJ cells. Treatment with SZE lowered COX-2 and 8-OHdG and increased MT significantly (p < 0.05) in UV-induced BJ cells. SZE treatment with a concentration of 25 μg/mL reduced COX-2 and 8-OHdG and increased MT was the most active than other concentrations.

### Effect of SZE concentrations toward apoptosis percentage in UV-induced BJ cells

**Figure 5:**
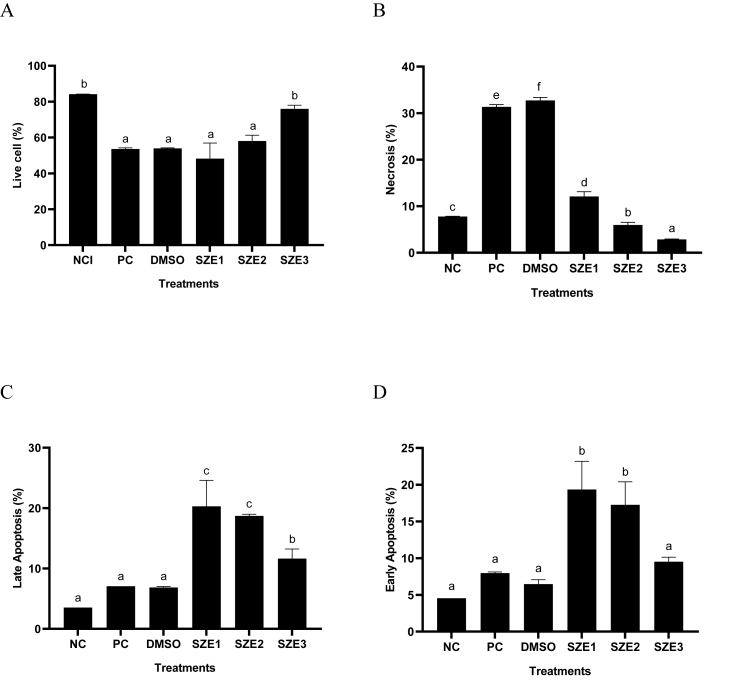
Effect of SZE Concentrations toward Live, Necrosis, and Apoptosis Cells in UV-Induced BJ Cells. ∗(A) live cells, (B) necrosis cells, (C) late apoptosis, and (D) early apoptosis. Differences of statistical significance (p < 0.05) among the treatments were identified using distinct superscript signs according to the Mann–Whitney test. NC: negative control; PC: positive control (UV-induced); DMSO: PC + DMSO 1 %; SZE1: PC + SZE 6.25 μg/mL; SZE2: PC + SZE 12.5 μg/mL; SZE3: PC + SZE 25 μg/mL.

Figure 5 shown the results of SZE different treatments toward apoptosis in UV-induced BJ cells. The findings demonstrated that administration of UV led to a reduction in viable cells and triggered necrosis and apoptosis. Increasing the concentration of SZE treatment increased the percentage of live cells and decreased necrosis. The low concentrations of SZE (6.25, 12.5 μg/mL) were less active to increased the live cells, decreased necrosis and apoptosis cells.

## Discussion

UV light exposure can increase the likelihood of premature skin aging by the induction of DNA damage, degradation of ELN and COL, and death of fibroblast cells.^19^ The use of antiaging chemicals for the treatment of skin aging issues is sometimes associated with adverse long-term effects. Consequently, there is a demand for alternative natural substances that incorporate antiaging bioactives, thus ensuring long-term safety and efficacy. Snake fruit is a tropical fruit that has garnered significant attention in academic research due to its rich content of active chemicals whose effects as antioxidants and antiaging agents have been demonstrated in multiple investigations.^6^^,^^16^^,^^20^^,^^21^ In this research, LC-MS revealed that SZE contains natural compounds such as chlorogenic acid, epicatechin, apigenin, and naringenin (Figure 1). This result aligns with previous studies that found compounds such as polyphenols, flavonoids, chlorogenic acid, and caffeic acid in *S. zalacca*.^5^

The results of the cytotoxic test indicated that SZE did not have a toxic effect on BJ cells. This conclusion is supported by the observed percentage inhibition at the maximum dose, which was found to be 26.56% (Figure 1). Therefore, three concentrations were chosen based on their superior survivability levels, specifically over 90 %. These concentrations are 25, 12.5, and 6.25 μg/mL. The results of UV induction in BJ cells can significantly influence gene expression and protein levels measured in this study, as evidenced by the disparity in notation between NC and PC for each parameter. This finding aligns with previous studies that have demonstrated the activation of *MMP-1* and *COL1A1* by UV radiation.^22^ Another *in vitro* study that UV stimulation has the potential to elevate the levels of COX-2 and 8-OHdG proteins.^23^

The quantification results of gene expression indicate that the treatment with SZE leads to upregulation of the *MMP-1* gene and downregulation of the *COL1A1* gene in UV-induced BJ cells, as depicted in Figures 3A and 3B. The activity of the gene can be inhibited by the bioactive compound in SZE, specifically chlorogenic acid, which exhibits a high binding affinity for MMPs (−9.4 kcal/mol) *in silico*.^6^ To prevent damage to dermis collagen, it is necessary to limit the activity of *MMP-1*, which is involved in the destruction of extracellular matrix such as collagen and elastin. Other research reported that upregulation of the *MMP-1* gene is associated with upregulation of the *COL1A1* gene, which is involved in the synthesis of type 1 collagen.^24^

*FGF-2* has a crucial role in the regeneration of fibroblast cells. Nevertheless, the regulation of the *FGF-2* gene can be impeded by exposure to UV radiation. The findings demonstrated that SZE could enhance the expression of the *FGF-2* gene in BJ cells (Figure 2C). The concentration at which *FGF-2* gene expression is most effectively enhanced is 25 μg/mL. The other study showed that snake fruit possesses flavonoids that have been observed to enhance the production of *FGF-2* during the healing phase of traumatic ulceration in Wistar diabetic rats.^25^ It has been observed that flavonoids possess the ability to enhance cell proliferation and migration in mouse 3T3 fibroblast cells that have been damaged.^26^

The expression of the *GPX-1* gene is crucial in combating oxidative stress caused by the presence of ROS, particularly hydrogen peroxide (H_2_O_2_), which is generated as a result of UV exposure. It is necessary to enhance the expression of this gene to prevent further damage to the skin caused by ROS. The findings indicate that the administration of SZE at a concentration of 25 μg/mL leads to an upregulation of the *GPX-1* gene in UV-induced BJ cells (Figure 2D). The SZE content, specifically gallic acid, exerts an influence on this phenomenon. Multiple studies have demonstrated the efficacy of gallic acid in mitigating oxidative stress and replenishing antioxidant enzymes, such as catalase, superoxide dismutase, and *GPX-1*.^27^^,^^28^

ELN and COL are the primary extracellular matrix proteins that play an important role in maintaining the structural integrity of the skin. Both of these proteins exhibit a high degree of sensitivity to external stimuli, such as UV light. These findings align with the results of the study, indicating a significant difference in ELN levels between the UV positive control and the negative control. Administering SZE at 25 μg/mL could enhance the ELN level in UV-induced BJ cells (Figure 3A). Additional studies of *S. zalacca* peel indicate the presence of ferulic acid and proline, which can promote the production of COL and ELN. Additionally, cinnamic acid is found in the peel of *S. zalacca*, which enhances cell regeneration.^29^ Previous research proved that SZE could inhibit elastase activity by *in vitro* study with IC_50_ 19.71 μg/mL was categorized as very strong activity (Widowati et al., 2023).^3^

HAse is an enzymatic catalyst that exhibits a strong correlation with the aging phenomenon observed in the skin. The inhibition of the HAse enzyme is necessary due to its potential to degrade the hyaluronan structure responsible for tissue binding in the skin. The findings indicated that the administration of SZE 25 μg/mL effectively decreased the HAse levels in UV-induced BJ cells (Figure 3B). This finding aligns with previous studies that have indicated the presence of protocatechuic acid and ferulic acid components in SZE. The IC_50_ values for protocatechuic acid and ferulic acid toward HAse inhibition activities were found to be 107.77 μg/mL and 396.12 μg/mL, respectively.^30^

Skin inflammation caused by ROS can potentially accelerate the process of premature aging. COX-2, a protein involved in this process, produces prostaglandins that contribute to increased skin damage and an elevated susceptibility to skin cancer. UV light exposure can elevate the levels of COX-2 in BJ cells. Administering SZE at 25 μg/mL could reduce the levels of COX-2 in UV-induced BJ cells (Figure 4A). The flavonoid chemicals present in SZE are crucial in the inhibition of COX-2. Numerous investigations have substantiated this assertion through both *in vitro* and *in vivo* examinations.^31^, ^32^, ^33^

Biomarkers of oxidative damage and antioxidant levels in compounds can serve as therapeutic targets in the presence of ROS resulting from UV exposure. This study examined the use of 8-OHdG as a marker for DNA damage. In comparison to the negative control, the positive control exhibited a statistically significant elevation in the 8-OHdG level. The use of SZE can effectively decrease these protein levels (Figure 4B). The concentration of MT, a crucial endogenous antioxidant, exhibited a decline following exposure to UV radiation. However, it was observed that the MT level could be restored following treatment with SZE at 25 μg/mL (Figure 4C). The role of chlorogenic acid is significant in this pathway. Other studies have indicated the potential of chlorogenic acid in mitigating oxidative stress in DNA by modulating biomarkers such as 8-OHdG, total oxidant status (TOS), oxidative stress index (OSI), and malondialdehyde (MDA).^34^

**Figure 6:**
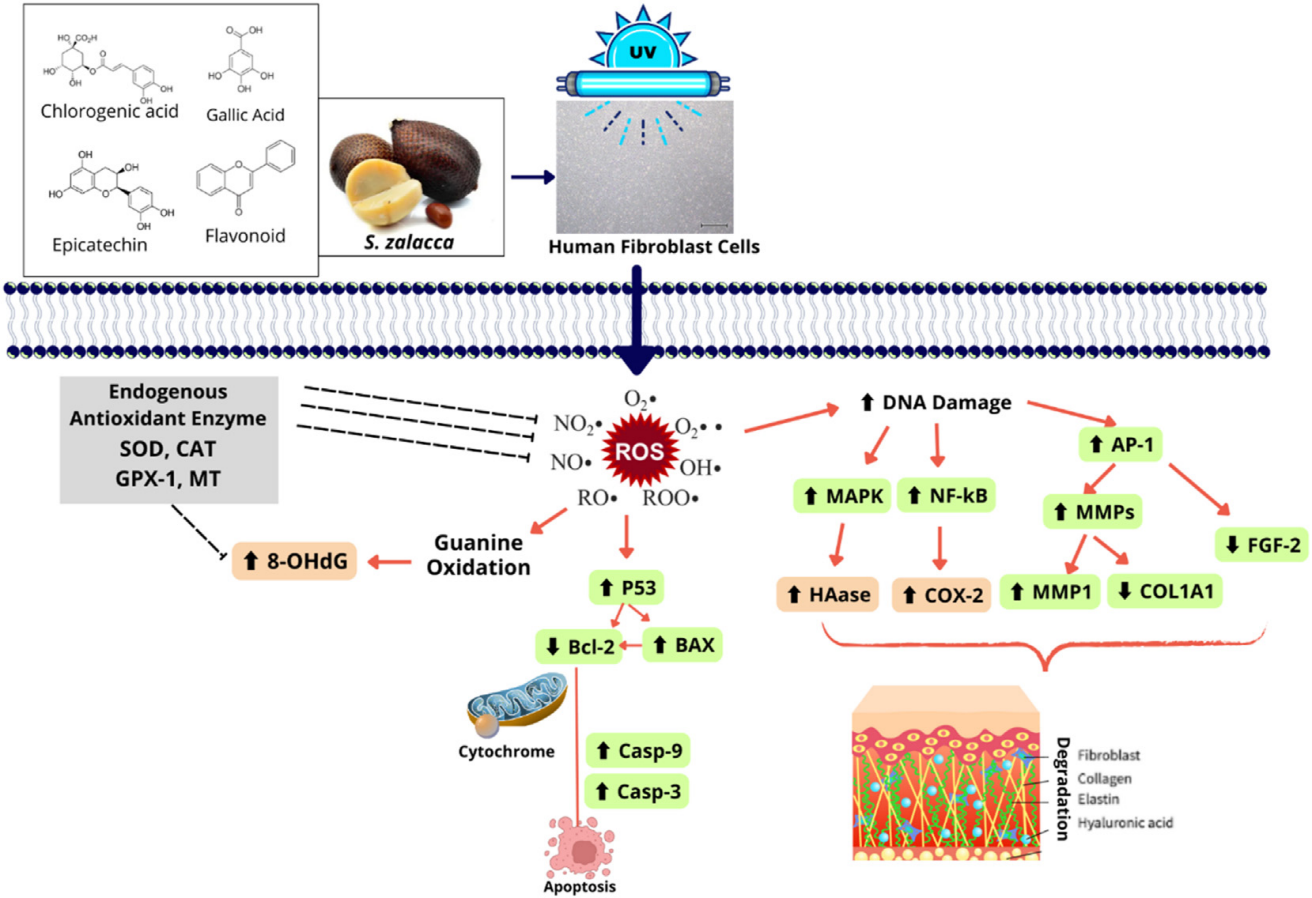
Proposed Mechanism of SZE as antiaging.

Premature aging can be regulated by apoptotic pathways in addition to antioxidant and anti-inflammatory mechanisms. UV radiation can produce ROS, triggering apoptosis in healthy fibroblast cells, resulting in the loss of their essential functions. The administration of SZE has been shown to effectively mitigate apoptosis and preserve the viability of UV-induced fibroblast cells (Figure 5). Consistent with previous studies, chlorogenic acid has been found to reduce apoptosis in UVB-induced human fibroblast cells.^18^ Therefore, the bioactive compounds in *S. zalacca* extract act synergistically to mitigate skin damage associated with premature aging This result research is achieved through the enhancement of gene and protein regulatory pathways and the amelioration of UV-induced apoptosis in human fibroblast cells. The proposed mechanism can be seen in Figure 6.

Exposure to UV light can increase the risk of premature aging in human fibroblast cells. UV exposure increases ROS in various forms such as H_2_O_2_, NO, and others that affect the regulation of genes and proteins related to aging. One of the effects is to cause DNA damage to the nucleus which results in chaotic regulation of signaling genes such as *MAPK, NF-kB,* and *AP-1*. This gene affects other genes such as *COL1A1*, *MMP-1*, and *FGF-2* which ultimately causes degradation of COL, ELN, hyaluronic acid, and fibroblast cells themselves which play a role in the strength and flexibility of the dermis. In addition, ROS also causes apoptosis in fibroblast cells through the regulation of the *P53* gene. Endogenous antioxidant activity can also be disrupted so that it is unable to capture ROS in cells. *S. zalacca* extract has bioactive compound components such as chlorogenic acid, gallic acid, flavonoids, and epicatechin which are antioxidants and antiaging so that ROS levels in cells can be reduced.

## Conclusion

SZE has potential as an anti-aging agent in UV-induced human skin fibroblast cells with the ability to increase the expression of *COL1A1, FGF-2*, and *GPX-1* and decrease the expression of the *MMP-1* genes. In addition, SZE also increased ELN and MT levels and decreased HAse, COX-2, and 8-OHdG levels in BJ cells exposed to UV. SZE can also maintain BJ cell viability and reduce apoptosis. Although these results indicate a protective effect against oxidative stress and collagen synthesis, this study did not directly trigger aging markers. Therefore, further studies are needed to confirm the anti-aging potential of SZE in the context of skin aging more broadly. The optimal concentration of SZE in this study was 25 μg/mL.

## Data availability

The data used to support the study are included in the article.

## Ethics Information

This study did not involve any human participants or animal subjects; therefore, ethical approval was not required.

## Authors contributions

WW: Conceptualization, Supervision, Writing – review & Editing. DD: Research Methodology. VV: Compile the data and statistical analysis. TLW: Conceptualization, Methodology, Supervision. FR: Investigation, Writing – review & Editing. FT: Methodology, Validation, Writing – Original Draft. PO: Formal analysis, Data curation. RT: Project administration, Data curation. FHZ: Writing – Original Draft, Formal Analysis, Visualization RA Methodology, Writing – Original Draft, Validation. DP: Investigation, Resources, Writing – review & Editing. WS: Project administration, Visualization, Resources. DSH: Resources, Data curation, Visualization. All authors read and approved the final manuscript. All authors have critically reviewed and approved the final draft and are responsible for the content and similarity index of the manuscript.

## Conflict of interest

The authors have no conflict of interest to declare.
