# Supplementary figures and images for: Salacca zalacca extract's antiaging effect on aging genes, protein levels, and apoptosis in UV-induced fibroblast cells
Source: J Taibah Univ Med Sci. 2025 Jun 9;20(3):349–58. doi: 10.1016/j.jtumed.2025.05.005 (PMC12180987; doi:10.1016/j.jtumed.2025.05.005)

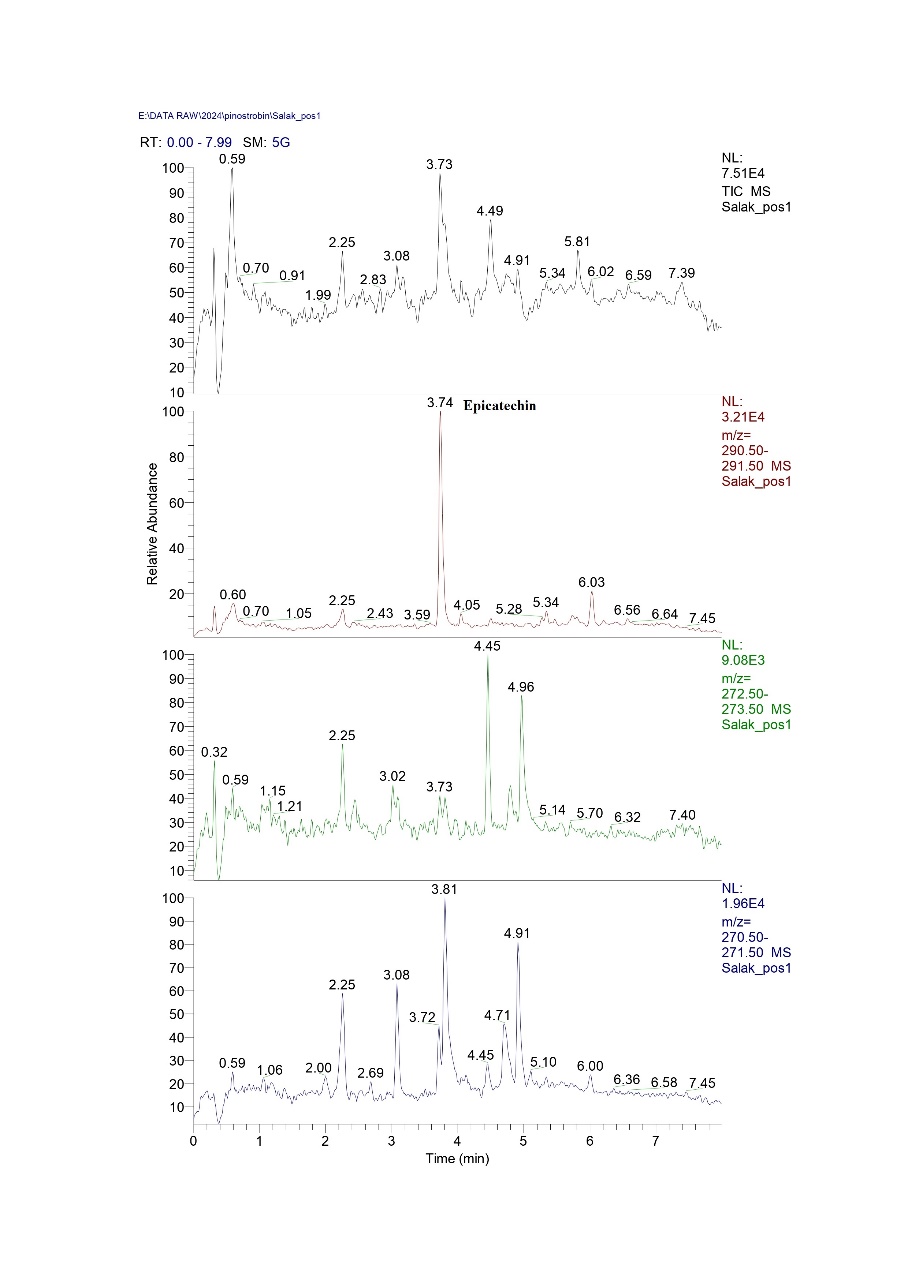

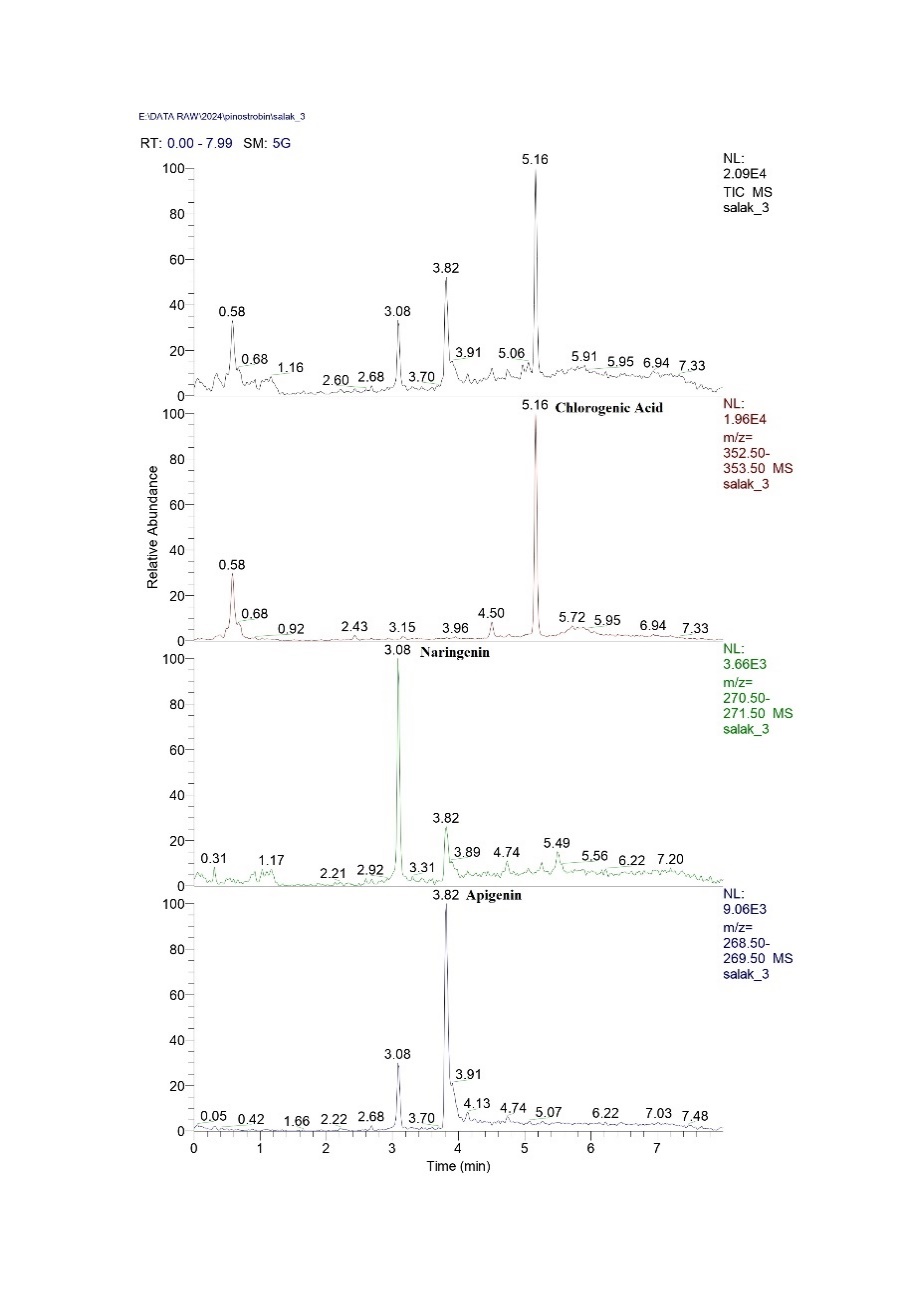


Supplementary Figure 1: LC-MS spectrum of SZE

Supplement: Multimedia component 1 [file mmc1.docx]
